# Artificial Intelligence Solutions to Increase Medication Adherence in Patients With Non-communicable Diseases

**DOI:** 10.3389/fdgth.2021.669869

**Published:** 2021-06-29

**Authors:** Aditi Babel, Richi Taneja, Franco Mondello Malvestiti, Alessandro Monaco, Shaantanu Donde

**Affiliations:** ^1^Leeds Teaching Hospitals NHS Trust, Leeds, United Kingdom; ^2^Medical Product Evaluation, Pfizer Ltd, Mumbai, India; ^3^Viatris, Rome, Italy; ^4^HEC, Paris, France; ^5^Viatris, Surrey, United Kingdom

**Keywords:** artificial intelligence, NCD, machine learning, big data, compliance, cardiovascular disease, digital health, patient empowerment

## Abstract

Artificial intelligence (AI) tools are increasingly being used within healthcare for various purposes, including helping patients to adhere to drug regimens. The aim of this narrative review was to describe: (1) studies on AI tools that can be used to measure and increase medication adherence in patients with non-communicable diseases (NCDs); (2) the benefits of using AI for these purposes; (3) challenges of the use of AI in healthcare; and (4) priorities for future research. We discuss the current AI technologies, including mobile phone applications, reminder systems, tools for patient empowerment, instruments that can be used in integrated care, and machine learning. The use of AI may be key to understanding the complex interplay of factors that underly medication non-adherence in NCD patients. AI-assisted interventions aiming to improve communication between patients and physicians, monitor drug consumption, empower patients, and ultimately, increase adherence levels may lead to better clinical outcomes and increase the quality of life of NCD patients. However, the use of AI in healthcare is challenged by numerous factors; the characteristics of users can impact the effectiveness of an AI tool, which may lead to further inequalities in healthcare, and there may be concerns that it could depersonalize medicine. The success and widespread use of AI technologies will depend on data storage capacity, processing power, and other infrastructure capacities within healthcare systems. Research is needed to evaluate the effectiveness of AI solutions in different patient groups and establish the barriers to widespread adoption, especially in light of the COVID-19 pandemic, which has led to a rapid increase in the use and development of digital health technologies.

## Introduction

### Non-communicable Diseases

Non-communicable diseases (NCDs), such as cancer, cardiovascular disease, chronic respiratory disease, and diabetes, are rising in prevalence due to multiple factors, including increased life expectancy, reduced premature mortality, and an increase in preventable risk factors (1). NCDs account for more than two-thirds of global deaths; cardiovascular diseases, in particular, account for about half of the deaths due to NCDs (1–3). Common NCDs are a major cause of disability (4), and pose a substantial economic burden to healthcare budgets and the welfare of nations (5).

### Medication Adherence

Medication adherence (defined as the extent to which a person's behavior regarding medication corresponds with agreed recommendations from a healthcare provider) is critical for achieving intended clinical outcomes in patients with NCDs. There are several ways to assess medication adherence; subjective measures can be useful for providing explanations about a patient's non-adherence, whereas objective measures may help to more precisely record an individual's medication-taking behavior (6). Data from US Medicare members with one or more NCDs (specifically diabetes, hypertension, and/or high cholesterol) demonstrated high rates of non-adherence to medications for these conditions; 76% were non-adherent to one of the three medicines, whereas 32% were non-adherent to more than one target medication class (7). Medication non-adherence is associated with multiple negative outcomes, including mortality and hospital admissions (8–12). A complex range of factors contributes to poor medication adherence: patient-related factors (such as health literacy, multimorbidity, and lack of involvement in the treatment decision-making process), physician-related (such as communication barriers or having multiple physicians providing care), and healthcare system–related (including limited access to care and lack of health information technologies) (13). Unfortunately, to date, even the most effective interventions for improving medication adherence have not resulted in large improvements in adherence or clinical outcomes (14). Although pharmacist-led interventions appear to be the most effective (15), interventions are often complex, involving numerous healthcare providers and multiple components (14). Effective adherence support thus requires a combination of interventions to optimize adherence at multiple levels, including the patient, the healthcare professional, and the healthcare system (16). As barriers to medication adherence are complex and varied, solutions to improve adherence must be multifactorial (13), and artificial intelligence (AI) technology is regarded to be a promising aspect of such interventions.

### Artificial Intelligence in Healthcare

There is no consensus on what constitutes AI, which usually refers to a computer mimicking intellectual processes that are characteristic of humans (e.g., the ability to reason, discover meaning, generalize, or learn from past experience) to achieve goals without being explicitly programmed for specific action. Generally, there are three types of AI (17): systems aimed at simulating human reasoning (and behavior) tend to be called “strong AI”; systems that can produce results similar to humans (but may use very different methods) are “weak AI;” and “in-between” systems are those informed or inspired by human reasoning. The latter one tends to be where most of the more powerful work is happening today in the industry.

AI includes the use of a computerized system (hardware or software) to model intelligent behavior with minimal human intervention which, in medicine, can fall under two branches; virtual (i.e., informatics and deep learning information) and physical (i.e., robot-assisted systems) (18). Within healthcare, AI has a range of potential uses, including aiding in the early detection, diagnosis, management, and treatment of medical conditions, improving patient engagement and increasing medication adherence, elderly assistance, health promotion, administering counseling, administrative activities, and even supporting education and learning for healthcare professionals (19–27). Since 2012, a rapid advancement in research in the field of healthcare–related AI (28) has resulted in a fast increase in the number of FDA-approved AI-based medical solutions (19), which is anticipated to continue growing in the future. Much of the literature focuses on diagnostic imaging and the main disease focus is neoplasms followed by nervous and cardiovascular diseases (29). A review of AI-based health-coaching systems used by patients with NCDs identified seven potential roles of these solutions; developing adherence, informing, motivating, reminding, preventing, building a care network, and entertaining (25). In this paper, we focus on how AI can be used to measure and increase medication adherence in patients with NCDs.

### Aim

The aim of our narrative review was to: (1) describe studies from the literature on AI and AI-assisted solutions that can be used to measure and increase medication adherence in patients with NCDs; (2) outline the benefits of using AI for increasing medication adherence; (3) discuss the barriers and challenges of the use of AI in healthcare; and (4) highlight research gaps and recommend priorities for future research.

## Methods

To identify topics of interest for this narrative review, we searched Pubmed, Embase, and Web of Science for relevant articles. We used search terms that could identify a wide range of topics, including artificial intelligence, machine learning, digital health, healthcare, disease management, smartphone applications, apps, drug reminders, reminder systems, adherence, medication adherence, drug adherence, compliance, non-communicable diseases, NCDs, and chronic disease, and search terms for specific NCDs, such as diabetes, stroke, and cardiovascular disease. We also reviewed the reference lists of relevant articles and previous reviews on similar topics to identify papers of potential interest. We focused on papers with NCDs as the main theme, especially ones focusing on adherence. The authors independently extracted information from the papers according to the aims, including the study methodologies, sample sizes, patient types, description of AI technologies, outcomes, and results. During meetings, the authors discussed the papers and selected appropriate examples for the narrative review.

## Current AI Technologies to Increase Medication Adherence in NCD Patients

In the following section, we describe some specific examples of how AI and AI-assisted technology can be used in NCD patients to measure or increase drug adherence.

### Mobile Phone Applications

AI smartphone applications (“apps”) have been evaluated as tools for assessing and encouraging medication adherence in a small number of studies. Labovitz et al. (30) developed an AI smartphone app to measure medication adherence in stroke patients taking direct oral anticoagulant therapy. The AI application used a neural network computer vision algorithm with the smartphone camera to visually identify the patient, the drug, and the confirmed ingestion. The software provided medication reminders and dosing instructions. In this 12-week randomized, parallel-group study, plasma drug concentration levels indicated 100% adherence in the intervention group that underwent daily monitoring via the AI platform, compared with 50% in the control group who received no daily monitoring. The absolute improvement of drug adherence was 67% in patients who were monitored by the AI app; 83.3% patients regarded the platform as “extremely good” when asked to rate the AI platform as a medication management tool and method to improve the doctor–patient relationship in a post-study questionnaire. However, the study was small (n=28) and ran for a relatively short period, highlighting the need for larger studies to determine whether substantial improvement in medication adherence can be maintained with such platforms over longer periods of time.

Most clinical trials currently use indirect and potentially inaccurate measures of adherence, such as pill counts and self-reported data. Bain et al. (31) evaluated a real-time monitoring method on a smartphone-based AI platform to assess adherence in a Phase II clinical trial of a 7-nicotinic receptor antagonist (ABT-126) in 53 patients with concurrent schizophrenia and cognitive impairment. The AI platform facial recognition, computer vision, and software algorithms helped to identify the patient and drug, and confirm ingestion in a 24-week trial. Findings showed a 17.9% higher adherence in the AI group vs. the control group who received modified directly observed therapy. The authors concluded that the AI platform could more rapidly detect non-adherence and predict future non-adherence compared with conventional methods.

### Reminder Systems

AI has also been used to deploy health communication to encourage adherence, for example, automated systems have been utilized to provide reminders to take medications. Brar Prayaga et al. (32, 33) assessed an SMS-based refill reminder solution using conversational AI, the “mPulse Mobile” in older patients with NCDs. A significantly higher medication refill rate was observed in the intervention group compared with the control group (who did not receive SMS reminders) (33). Similarly, the ChronologyMD project for Crohn's disease used a system of patient-sourced “observations of daily living” (ODLs) with computer-mediated AI support to enable patients to track their medication adherence, activity, and symptoms on a daily or hourly basis; this information was then automatically made available to providers to improve decision making (23). Patients reported that the AI components of the system enabled them to track, understand, and monitor their ODLs more easily and accurately while also helping them to remember to take their medications.

### AI for Patient Empowerment

AI has also shown indirect medication adherence benefits through patient support and empowerment. “Vik” is a chat-bot that was designed to empower patients with breast cancer and their relatives (34) via personalized text messages. It provided relevant, quality-checked medical information about breast cancer, its epidemiology, treatments, and side effects, as well as information about lifestyles, fertility, reimbursement, and patients' rights, and so on. The study showed that the more the participants chatted with Vik, the more observant they were when using a treatment reminder function, and the average compliance of patients using the medication reminder feature improved by more than 20%. Alternatively, robotic assistants may be another method to increase patient empowerment; AI robot assistants have been shown to be helpful in the self-management of diabetes and insulin control in children (35–37).

### AI in Integrated Care

Integrated care programs are generally referred to as care systems that aim to foster coordination within and between healthcare organizations and healthcare professionals, with the goal to improve clinical outcomes and the experience of patients. These programs can enhance the care and treatment of patients with NCDs and are also recommended for complex patients, such as those with multimorbidity (defined as the presence of more than one concurrent NCD) (38). A systematic review (39) reported beneficial effects of integrated care for adults with NCDs on several outcomes, including reduced mortality, reduced hospital admissions and re-admissions, and improved adherence to treatment guidelines (including adherence to treatments or diets and/or provider adherence to guidelines). Integrated care involves several components that could be supported by AI-assisted technology, including multidisciplinary networks, sharing of information between healthcare providers and using electronic patient records and computerized clinical charts, strengthening patients' self-management and self-efficacy, shared decision making, and the use of individualized care plans, among others (38). Natural language processing and cognitive computing AI methods could enable medical data managers to organize and mine data from unstructured electronic medical records automatically. AI-assisted technology could also be used to optimize prescriptions by prioritizing medications that match the insurance/preferred pharmacy of the patients and check drug–drug interactions, and so on. AI has already been shown to be useful for medication reconciliation, which is a procedure often used to reduce medication errors. An AI-assisted tool was developed to improve medication reconciliation by engaging the patient and healthcare providers as a team via a tablet-based tool (40). The AI component was found to help the patients recognize their own medications and report discrepancies for the clinicians to review (40), consequently resulting in improved medication accuracy and reduced medication errors.

### Machine Learning and Big Data Analytics

One of the major contributions that AI-assisted technologies has had in recent years in NCD management is through machine learning and big data analytics. A systematic review of literature on AI highlighted that machine learning is currently the most commonly used AI technology in healthcare (28). However, this field is still in its infancy; there are currently <100 FDA-approved AI/machine learning-based medical devices and algorithms (19), which are constantly updated on an online database (41). Although many relate to computer-assisted diagnosis and clinical decision support systems, there are several interesting examples in the scientific literature of how machine learning has been used to measure, predict, and increase medication adherence in NCD patients. For example, machine learning has been used to identify factors related to adherence to nicotine replacement therapy (42) as well as diabetes and Crohn's disease medication (43, 44), and asthma self-management (45). Koesmahargyo et al. (46) assessed the accuracy of medication dosing data to predict medication non-adherence through machine learning in a study using a large sample of participants from a range of clinical trials, who were observed via a smartphone application that used videos of the patients taking their prescribed medication. The real-time measurement of dosing was able to dynamically predict medication adherence with high accuracy over the trial period as well as over the subsequent day and week (46). Machine learning models were also found to be effective in identifying the key variables to understand the adherence levels of hypertensive patients (47) and to even predict adherence to lifestyle patterns, such as the Mediterranean diet (48).

Machine learning methods can also be extended to examine the effect of medication adherence on clinical outcomes. For example, in a study using data from more than 30,000 patients with type 2 diabetes mellites, machine learning was used to examine how adherence to oral hypoglycemic medications was associated with reduced hospitalization (49). In another study, an AI framework that was designed to learn from clinical data was able to improve patient outcomes by 30–35% compared with treatment-as-usual (50).

Big database analysis techniques have also been utilized for measuring the effectiveness of interventions, for example, investigating adherence as a clinical outcome (51). Importantly, the benefit of machine learning is that combining a magnitude of data sources into a comprehensive AI dataset can help to optimize treatment and adherence (52). Considering the multifaceted components of integrated care, these techniques may become increasingly useful for optimizing medication adherence in NCD patients with complex conditions such as multimorbidity, especially for delivering precision medicine (53).

## Benefits of AI to Improve Medication Adherence

### Assessing Adherence Levels

As outlined, AI has shown promising results in both measuring adherence levels and improving medication adherence. Currently, assessing medication adherence and its underlying influencing factors can be challenging for physicians and researchers. There is a multitude of direct and indirect, as well as objective and subjective methods available for measuring these; patient self-report, electronic measures, and pharmacy refill and claims data are currently the most commonly used measures in research, routine practice, and epidemiological and intervention studies (54). However, self-reported measures have only a weak-to-moderate correlation with, for example, prescription refills and electronic measures. A combination of these methods is anticipated to be a useful approach to increase the validity and reliability of adherence measurements (54), which could be achieved utilizing AI methods, especially machine learning methods.

### Increasing Adherence

As described in the examples above, several trials assessing AI-assisted technologies have demonstrated increased adherence rates in patients using the technology vs. conventional care (30, 31, 33). Increased medication adherence is associated with improved clinical outcomes in NCD patients. For example, adherence to cardioprotective medications in patients with diabetes and ischemic heart disease was shown to be associated with lower all-cause mortality (9), and adherence to statins and beta-blockers in patients with acute myocardial infarction was associated with a lower risk of long-term mortality (median of 2.4 years) (11). Furthermore, a systematic review concluded that hospital admissions associated with non-adherence to medication are a common problem, especially in relation to cardiovascular medicines (8). A further meta-analysis demonstrated a 21% reduction in long-term mortality risk with good medication adherence in comparison to medication non-adherence, and a 17% higher risk of all-cause hospitalization associated with non-adherence in older adults (12). Clinical outcomes may also be affected, for example, diabetic patients who do not adhere to their medication regimen have poorer clinical outcomes, such as higher glycosylated hemoglobin levels, systolic and diastolic blood pressure, and low-density lipoprotein cholesterol levels (10), as well as a higher risk of all-cause mortality and all-cause hospitalization (10). Medication non-adherence also places a significant cost burden on healthcare systems; a systematic review estimated the annual adjusted disease-specific economic cost of nonadherence as US$949 to US$44,190 per person (in 2015) and the costs attributed to all-cause non-adherence ranged from US$5,271 to US$52,341 (55). Increasing medication adherence is also important for patient-related outcomes as interventions have shown that increasing adherence can lead to a better knowledge of medications as well as improvements in quality of life, physical function, and symptoms (56).

### Time Saving

In clinical practice, patients' subjective estimates of adherence might be inaccurate, and physicians may not have time to explore and address the underlying reasons behind non-adherence, especially because a complex range of factors can influence it (13, 57). Using AI-assisted technologies may, therefore, help to free up the time of healthcare providers for essential clinical activities and more in-depth disease- and treatment-related communication with patients. It has been suggested that AI could enable a more accurate understanding of a patient's medication adherence so that physicians can dedicate more time to judgment and emotional intelligence for developing personalized strategies to optimize adherence (22). Furthermore, in light of the ongoing COVID-19 pandemic, it must be noted that AI-assisted interventions and monitoring may be essential for NCD management in periods where face-to-face healthcare is restricted. For example, the pandemic has led to a substantial decrease in admissions for acute cardiovascular disease, reductions in the number of procedures, and longer delays between symptom onset and hospital treatment (58). Although limited research is available on how the pandemic and infection control measures have specifically affected medication adherence in NCD patients, one systematic review reported a significant failure of inflammatory bowel disease patients to adhere to therapies during the COVID-19 pandemic (59). Furthermore, some, but not all, studies have reported higher antibiotic prescribing rates in remote primary care consultations compared with face-to-face settings (60). AI, with its potential to assess adherence via smartphones and tablet-based monitoring systems, as described above, may thus provide additional benefits through remote monitoring of patients and increasing communication with healthcare practitioners during pandemic times.

### Scientific Discovery

AI technologies, especially machine learning and big data analytics, have another important advantage; these are believed to advance scientific discovery and our knowledge on intricate factors underlying adherence and, consequently, our understanding of methods to improve it. Processing extremely large and complex data sets in healthcare can unlock novel insights and accelerate breakthroughs in medicine. AI technologies can assist healthcare professionals and researchers in quicker data processing compared with traditional methods. This might be an essential element for providing precision medicine, which may be particularly useful for the management of older patients with complex conditions, such as multimorbidity (53).

## Challenges of Using AI in HealthCare

### Patient Characteristics

The use of AI for measuring or increasing medication adherence may lead to inequalities in healthcare between individuals as the characteristics of users can impact the effectiveness of an AI tool. For example, a study on the efficacy of an SMS-based refill reminder solution using conversational AI in patients with the chronic disease found that race, ethnicity, language, age, and social determinants of health affected the level of engagement with the AI solution to improve refill adherence (32). A consumers' survey (61) suggested that technological, ethical, and trust factors, as well as regulatory concerns, significantly, contribute to the perceived risks of using AI applications in healthcare. Specifically, technological concerns were the most significant, particularly perceived performance anxiety (i.e., the user's perception that the IT system will malfunction or not work as intended) and communication barriers (i.e., the extent to which the user thinks that the AI device might reduce human aspects of relations in the treatment process). Furthermore, AI-led chat-box services in healthcare can be affected by the user's trustworthiness and perceived poorer IT skills and dislike for talking to computers (62). It is also worth considering that many of the factors that affect a user's ability and willingness to engage with AI technologies may also be factors that are also independently related to non-adherence. A systematic review in older patients identified three types of barriers to adherence: patient-related factors (sociodemographics, psychosocial profile, comorbidities, cognitive ability, and health beliefs); drug-related factors (number of drugs taken, adverse effects, and administration regimens); and other factors (patient–prescriber relationship, access to medication, and social support) (57). Thus, it is essential that future research investigates whether patients who have a high risk of non-adherence are able to engage well with AI-assisted interventions to increase adherence, or whether the use of such technologies will lead to further inequality in healthcare between individuals.

### Concerns About Depersonalization

Another barrier to the use of AI in healthcare is the potential concern from patients, relatives, and healthcare professionals that it may “depersonalize” medicine. This may be more pronounced in certain demographic groups; for example, in the field of AI and gerontechnology, there are concerns that algorithm-based standardization and automization may lead to the depersonalization of care, that the care relationship may be dehumanized through automatization, and that minority groups may experience discrimination through generalization (63), consequently challenging the “4p medicine” (predictive, personalized, preventive, and participatory) patient-centered model of care (63). There is also a skepticism toward automation, and the perceived risk stems from delegating control to machines and their respective control mechanisms. A systematic review of adherence interventions also reported that face-to-face interventions were more effective than interventions delivered in other ways, such as via a computer, telephone, and/or text message (15). Although such challenges apply to a range of disorders and different areas of healthcare, they are relevant also to the theme of medication adherence and NCDs. Importantly, as persons age, their risk of having more than one chronic condition (also known as multimorbidity) increases and this is often associated with polypharmacy and related negative outcomes, such as adverse drug reactions. AI technologies that monitor adherence may provide important opportunities for NCD patients on polypharmacy. It has been suggested that patients with multimorbidity benefit from integrated care models with regular monitoring and assessment by a team of specialists (38). Thus, the above-mentioned concern about depersonalized care may be especially relevant to complex patients with multiple comorbid conditions. However, if applied appropriately, AI-assisted technology has the potential to enhance the 4p model by liberating physicians from time-consuming tasks and potentially inaccurate methods for measuring subjective reports of adherence, thus providing them with more time to make emotional connections with their patients, increasing shared decision making, and enabling them to address individual reasons underlying non-adherence. AI is unlikely to replace physicians at the bedside, but can provide useful opportunities to enhance patient–physician communication; for example, the AI-assisted medication reconciliation tool described above (Long et al.) engaged patients and healthcare providers as a team by enabling patients to report medication discrepancies for their clinicians to review (40). The use of technology has been suggested as an essential component of integrated care models for NCD patients, especially those with multimorbidity (38).

### Infrastructure, Training, and Cost Effectiveness

The success and widespread use of AI technologies will depend on data storage capacity, processing power, and other infrastructure capacities within healthcare systems. For example, there may be challenges in migrating data across electronic and paper health records. Data and results must be understandable by healthcare professionals and, in some cases, patients. Healthcare systems should also support employees and the adoption of AI solutions by providing training courses to employees. However, less than half of healthcare insiders report that AI training is provided to their employees (64) and it was suggested that “Building an AI-ready workforce requires a wholesale change in the approach to training and how to acquire talent. Having people who understand how AI can solve big, complex problems is critical” (64). This may also help to reduce the risk of “AI-washing,” that is, where developers add an “AI” label to software platforms with basic algorithms to excite and exploit buyers, including healthcare systems and individual practitioners. Therefore, training employees at multiple levels of the healthcare system will result in better evaluation of the efficacy of an AI solution so that healthcare professionals can consider the long-term potential to improve patient outcomes and weigh up the cost-effectiveness.

A recent systematic review highlighted the lack of research thoroughly addressing the economic impact assessment of AI in healthcare within the current literature (65). Nevertheless, the costs associated with developing and initiating AI interventions may be expensive and this may be a relevant barrier to the use of these technologies in healthcare research and practice. As mentioned previously (55), medication non-adherence is associated with substantial increases in patient costs, so it is of utmost importance to establish whether AI and AI-assisted interventions actually increase or decrease the costs associated with non-adherence in future, well-designed cost-effectiveness studies.

### Ethical Issues

There is a myriad of ethical concerns that create barriers to the use of AI in healthcare (18, 63, 66–70). Who owns the data that is being mined during machine learning and big data analytics? Have privacy issues been addressed and have adequate steps been taken to ensure the safety and confidentiality of patient data? Are all users aware of regulations and guidelines for using AI-generated data and results? Will the use of AI lead to further inequalities in healthcare, for example, according to racial or economic factors (70)? There may be racial and ethnic differences regarding obstacles for participating in the donation of biospecimens for research (71), which may, in turn, have important implications for machine learning. In the field of NCDs, there are racial inequalities in terms of NCD risk factors as well as treatment issues, including adherence to medications for common NCDs, for example, antidiabetic, antihypertensive, or antihyperlipidemic drugs (72). Furthermore, given the highly sensitive nature of patient medical records, three quarters of healthcare insiders have concerns that AI could threaten the security and privacy of patient data (64). Particular measures should be taken to address the ethics of gerontechnology because the incidence of NCDs, the number and complexity of diseases, and multimorbidity increase with age, and are associated with polypharmacy (63, 73). Attention should be taken with regard to technologies that have disciplinary elements (i.e., direct or indirect disciplining by forcing patients to adapt to the standards set by the AI). Innovations that initially appear to empower the patient and improve their well-being may also turn into a means to achieve goals that are not in their primary interest (63). Thus, the development and use of AI in healthcare research and practice require careful consideration of ethical principles, risks and benefits, privacy, access and usability, and data management (68). A recent publication (66) proposed 20 critical questions that should be addressed whilst creating a framework for AI research, which can help to identify common pitfalls that may undermine machine learning and AI-based applications in health, centering around transparency, reproducibility, ethics, and effectiveness.

## Current Research Gaps and Future Directions and Research

AI in healthcare is still in its infancy. To date, the FDA has approved <100 AI/machine learning-based medical devices and algorithms (19, 41) for any type of application. The most rapid advancements in healthcare–related AI have occurred since 2012 (28), with the most common publications focusing on cancer, coronary artery disease, chronic kidney disease, prostate cancer, and diabetes mellitus (28). However, research within this field in low- and middle-income countries is still substantially lacking (28). Furthermore, despite the fact that older people are the primary sufferers of NCDs, most healthcare–related AI research has focused on children (28) and, therefore, more research on the efficacy and ethics of gerontechnology is needed (74, 75).

Kardas et al. (76) highlighted that the use of big data to study patient adherence is hindered by three major barriers in terms of standardizing data for analysis. They recommended that there should be a standard format for the data collected in big data databases, sound metrics for processing of the raw data, and commonly agreed standards of presentation of adherence measures being assessed within big data. Importantly, according to a recently proposed framework (66), future AI research in healthcare should consider issues of transparency, reproducibility, ethics, and effectiveness.

## Limitations

In this narrative review, we sought to provide an overview of the current status of AI in the assessment and improvement of medication adherence in patients with NCD. However, a few limitations must be discussed. Firstly, we did not conduct a systematic review of the literature because the literature contains only a few studies on the effectiveness of AI for measuring or improving medication adherence. Further, there is a large range of study designs and methodologies, which made it more appropriate to conduct a narrative review to provide an overview of this diverse body of literature. Many studies have small sample sizes and short durations, making it difficult to make firm conclusions about the effectiveness of some technologies for increasing adherence. Additionally, we believe that the ongoing COVID-19 pandemic has created a substantial shift in the characteristics of care delivery and research, which may affect future interpretations of our descriptions and conclusions. The use of AI in the field of medication adherence during the COVID-19 pandemic was not the focus of our paper, and the currently available literature does not reflect the potentially rapid advancement of AI-assisted technology development likely to occur as a result of the pandemic.

## Conclusions

The use of AI may be key to understanding the complex interplay of factors that underlie medication non-adherence in NCD patients. AI and AI-assisted interventions aiming to improve communication between patients and physicians, monitor drug consumption, and empower patients, and ultimately increase adherence levels may lead to better clinical outcomes and increase the quality of life of NCD patients. However, research on this topic is still sparse and a wide range of challenges remain, especially within the development and evaluation of AI solutions regarding their effectiveness in different patient groups and the barriers to widespread adoption.

## Author Contributions

AB and SD: made substantial contributions to the conception of the manuscript. AB: searched and critically appraised the literature for this review and a scientific writer drafted the manuscript. AB, RT, AM, FM, and SD: made substantial contribution to the interpretation of the data from the literature and critically revised the manuscript. All authors read and approved the final version.

## Conflict of Interest

RT is an employee of Upjohn, a division of Pfizer. FM and SD are full-time employees and stockholders of Viatris. The writing of this paper was commercially funded by Viatris, UK, in the form of a payment for professional scientific writing support to Oliba, Rome, Italy. No products or services of Viatris, UK, have been discussed or promoted within this manuscript. The remaining authors declare that the research was conducted in the absence of any commercial or financial relationships that could be construed as a potential conflict of interest.
